# *In vitro* assessment of antibacterial and biocompatibility properties of a poly-ε-lysine and hyaluronic acid contact-killing coating to prevent prosthetic joint infection

**DOI:** 10.1371/journal.pone.0340632

**Published:** 2026-01-30

**Authors:** Julia L. van Agtmaal, Anniek M.C. Gielen, Sanne W.G. van Hoogstraten, Laura C.W. Peeters, Aghilas Akkache, Rajendra Kasinath, Nihal Engin Vrana, Nick R.M. Beijer, Tim J.M. Welting, Cynthia Calligaro, Jacobus J.C. Arts

**Affiliations:** 1 Department of Orthopedic Surgery, Research Institute CAPHRI, Maastricht University Medical Center, Maastricht, the Netherlands; 2 National Institute for Public Health and the Environment (RIVM), Bilthoven, the Netherlands; 3 Institute of Biodiversity and Ecosystem Dynamics (IBED), University of Amsterdam, Sciencepark, Amsterdam, the Netherlands; 4 SPARTHA Medical, 1 Rue Eugène Boeckel, Strasbourg, France; 5 DePuy Synthes Biomaterials, Warsaw, Indiana, United States of America; 6 Orthopedic Biomechanics, Department of Biomedical Engineering, Eindhoven University of Technology, Eindhoven, the Netherlands; Advanced Materials Technology Research Institute, National Research Centre, EGYPT

## Abstract

Prosthetic joint infection (PJI) is a major adverse outcome following total hip and knee arthroplasties. With the rise of antimicrobial resistance, there is a risk of therapeutic insufficiency in treating PJI. This study determined the potential of a poly-ε-lysine and hyaluronic acid (PEL-10/HA144-1) contact-killing coating as a promising prevention of bacterial attachment. A broader development perspective was integrated through a Safe-by-Design approach by adding relevant *in vitro* tests for implant-host interactions. The PEL-10/HA144-1 coating was deposited on titanium alloy Ti6Al4V (Ti) and ultra-high-molecular-weight-polyethylene (UHMWPE). A combination of the ISO 22196, ASTM E2180-18, and JIS Z 2801 testing standards using *Staphylococcus aureus* and *Escherichia coli* demonstrated a bactericidal effect of the coating, up to a 5-log reduction compared to uncoated samples. A bacterial adhesion test showed a decrease in adherent bacteria up to 4-log after 4 h, and up to 5-log after 24 h between coated and uncoated samples. Saos-2 human osteoblast-like cells and L929 mouse fibroblasts exposed to 72 h extracts of the coating for 24 h showed *in vitro* cell viability of >70%, indicating no cytotoxicity according to ISO 10993−5. Furthermore, while initial osteoblast attachment to the coating appeared challenging, increased proliferation and metabolic activity over time were observed. After 14 and 21 days, no reduction in osteogenic marker expression was found on the coated samples compared to the uncoated samples. Overall, the PEL-10/HA144-1 coating reduced bacterial adhesion, was not cytotoxic to mammalian cells*,* and supported osteoblast function *in vitro*, making it a promising technique for future implantable orthopedic applications.

## Introduction

Total hip arthroplasties (THA) and total knee arthroplasties (TKA) successfully relieve pain and restore joint function in patients suffering from arthritis [1]. THA and TKA incidence have increased significantly and are expected to rise further due to an increasingly ageing population and other risk factors like obesity [2–4]. Prosthetic joint infection (PJI) following primary TKA and THA arises in 1–2% of the surgeries and is a leading cause of primary THA and TKA failure [4–7]. For revision joint arthroplasty, infection rates are up to 40% [5]. Delayed healing, inadequate functional outcome, decreased quality of life, and increased mortality occur as accompanying adverse outcomes, leading to increased healthcare costs [6,8,9]. Most PJIs are caused by gram-positive bacteria, primarily *Staphylococcus aureus (S. aureus)* and *Staphylococcus epidermidis* [7,8]. However, gram-negative bacteria like *Pseudomonas aeruginosa* and *Escherichia coli (E. coli)* can also be involved [7,10]. These bacteria form biofilms, dense bacterial accumulations encased in a protective extracellular matrix, shielding them from the immune system and antibiotics, contributing to persistent infection [11]. Unfortunately, the widespread overuse of antibiotics has accelerated the rise of antimicrobial resistance (AMR), increasing the risk of therapeutic insufficiency for PJI patients [8,12,13]. PJI poses an ever-growing threat, and in addition to new antibiotics, new material technologies are urgently required to prevent bacterial attachment and biofilm formation on implant surfaces, reducing the occurrence of PJI.

In the case of PJI and biofilm formation, prevention is better than cure [10]. Innovative surface modifications and antimicrobial coatings are being developed to prevent PJI development by hindering initial bacterial adhesion, biofilm formation, and maturation. Antimicrobial techniques can be subdivided into three major groups: anti-adhesion, contact-killing, and release-killing or leaching [8,14]. Total knee and hip joint implants are composed of medical-grade polymers and metals; thus, a coating against PJI must cover both types of substrates. Commonly used orthopedic biomaterials are titanium alloys Ti6Al4V (Ti) and ultra-high-molecular-weight-polyethylene (UHMWPE) [15–17]. Coatings frequently fail to properly adhere to polymeric surfaces, while these parts are highly susceptible to biofilm formation [16]. The incorporation of an antimicrobial functionality on an implant is primarily assessed based on its antimicrobial effect. However, it also influences the overall performance of the implant. Such material modifications often affect various unintended biological processes, impacting implant success [18]. Identification of these biological processes early in the development process is one of the first steps in the Safe-by-Design (SbD) principles, and is essential for the implant’s success and to reduce the risk of common adverse outcomes [18]. For orthopedic implant technologies, the prevention of bacterial adhesion, enabling osseointegration, and immune acceptance are crucial for successful implementation [18].

For this study, a novel polyelectrolyte antimicrobial contact-killing spray coating has been developed to prevent bacterial adherence and biofilm formation. This coating is based on self-assembling supramolecular microstructures of polycationic poly-ε-lysine (PEL-10) and polyanionic Hyaluronic acid (HA-1). Due to PEL’s positive charges, electrostatic interactions occur with the negatively charged components of the bacterial cell membrane [19–22]. In both gram-positive and gram-negative bacteria, this interaction results in a disrupted cell membrane, making PEL penetrate the bacteria and inhibit metabolic pathways critical for bacterial survival [19,21,22]. So far, bacteria are unable to develop resistance to PEL due to the physical nature of the mode of action [20,23]. The second component of the coating, HA-1, is known for promoting wound healing, being biocompatible, and possessing antifouling properties, which reduces bacterial attachment [24]. For coating application, a device for spraying a functional coating of supramolecular structures (SPARTHA protect|ION, patent number WO2025124895A1) has been manufactured [25,26]. Its conical flow channels enable controlled layer-by-layer spraying of polycation and polyanion solutions to create functional supramolecular coatings.

This study evaluated the potential of a PEL and HA spray-coating (PEL-10/HA144-1) for orthopedic implants to prevent bacterial attachment while maintaining biocompatibility. The PEL-10/HA144-1 coating was applied to Ti and UHMWPE substrates using the SPARTHA protect|ION and characterized. The *in vitro* antimicrobial activity was assessed according to ISO 22196, ASTM E2180-18, and JIS Z 2801 [27–29] for regulatory compliance, and using a bacterial adhesion test. Additionally, *in vitro* cell viability and toxicity were assessed following ISO 10993−5, and osteoblast-like cell adhesion, proliferation, metabolic activity, and osteogenic marker expression were tested to evaluate implant-host tissue interactions. A broader scope of tests was used to assess the antimicrobial and biocompatible nature of the coating, in alignment with the SbD framework.

## Methods

**Fig 1:**
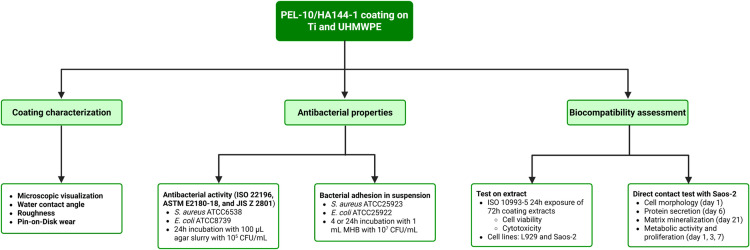
Schematic overview of the methodology. The poly-ε-lysine and hyaluronic acid (PEL-10/HA144-1) coating is sprayed on titanium (Ti) and ultra-high-molecular-weight-polyethylene (UHMWPE). Coating characterization, antibacterial properties, and biocompatibility were assessed in parallel. All bacteria are American Type Culture Collection (ATCC) strains. Mouse fibroblasts (L929, ATCC cell line CCL 1, NCTC clone 929) and human osteoblast-like cells (Saos-2, CLS, CLS 300331, epithelial-like) were used for biocompatibility assessment.

A schematic overview of the methods is visualized in Fig 1.

### Substrates

The Ti alloy was Ti6Al4V ELI (DePuy Synthes, Warsaw, Indiana, United States, or Acnis International, Chassieu, France), conforming to ASTM F136 standards. All samples were cut from bar stock using wire EDM, grit blasted with alumina 25 grit to achieve a consistent surface roughness of ~3 µm Ra, or sand-blasted, and cleaned in deionized (DI) water. The UHMWPE substrate was GUR 1020 cross-linked UHMWPE (DePuy Synthes, Warsaw, Indiana, United States). UHMWPE samples were machined from bar stock and washed in DI water. All samples had a diameter of 13.9 mm and a thickness of 5 mm. All samples were high-pressure steam-sterilized at 121°C for 15 minutes.

### Coating characterization

#### Coating process.

All samples were sonicated for 5 min in 70% ethanol and dried at room temperature. PEL solution was prepared at 10 mg/mL (PEL-10), and HA solution was prepared at 1 mg/mL (HA144−1; batch number 144) in Tris-NaCl buffer (20−20 mM) at pH 7.4. Per 1 cm^2^, 125 µL of each solution was applied. These solutions were sprayed onto one side of the samples using the SPARTHA protect|ION, creating a thin coating based on the electrostatic interaction between the positively charged PEL-10 and the negatively charged HA144−1. The samples were dried at room temperature before coating the other side. The samples were UV light sterilized on both sides for 30 minutes.

#### Coating visualization.

Poly-L-lysine (PLL) was conjugated with fluorescein isothiocyanate (FITC) to visualize the coating on the substrate. The samples were incubated in a PLL-FITC solution for 15 min and washed in Tris-NaCl (10–150 mM) buffer at pH 7.4. The stained coating was observed using a Zeiss LSM 710 confocal laser scanning microscope.

Three PEL-10/HA144-1 coated and uncoated Ti samples were mounted on aluminum stubs and imaged using SEM (Jeol JSM-IT200 InTouchScopeTM, Germany).

#### Water contact angle.

A 2 µL droplet of Milli-Q water (resistivity of 18.2 MΩ· cm) was put on each sample with a probe (OCA30 goniometer, DataPhysics instruments, Germany), and the water contact angle (WCA) was measured for at least 60 seconds (OCA20 software, DataPhysics instruments, Germany). The WCA was measured in three locations per sample, on three samples per experimental group.

#### Roughness & pin-on-disk wear test.

In the pin-on-disk (POD) test (Thelkin GmbH, Winterthur, Switzerland), a PEL-10/HA144-1 coated and uncoated polyethylene pin moved in a 10 × 10 mm square pattern relative to a cobalt-chrome (CoCr) disk. A Paul loading cycle was applied at 330 N and 1.6 Hz over three intervals of 0.33 million cycles, totaling 0.99 million [30]. Gravimetric measurements were taken using a Mettler XP205 balance, and non-contact interferometry was conducted pre- and post-test with a Zygo Interferometer. Wear rates were calculated using a best-fit linear regression, excluding the initial cycle data point. Roughness was measured during this test as the arithmetic mean height (Sa) in µm.

### Antibacterial properties

All experiments were conducted in a sterile environment. Furthermore, all reagents in this study were subjected to high-pressure steam sterilization at 121 °C for 15 minutes. For statistical significance, all experiments were repeated three times.

#### Cell lines and preparation.

All bacterial stocks were stored in 20% glycerol at −80 °C. Overnight axenic cultures were prepared of bacterial strains *S. aureus* (ATCC25923 and ATCC6538) and *E. coli* (ATCC25922 and ATCC8739) on blood agar plates (Thermo Scientific, R01217) at 37 °C. From this axenic culture, 2–3 colonies were cultured (18–20 h, 37 °C, 200 rpm) in 5 mL tryptic soy broth (TSB; Sigma-Aldrich, 22092). Bacteria were resuspended in diluted nutrient broth (NB, 500x diluted with sterile DI water; Sigma-Aldrich, 70149) for the antibacterial activity test, or Mueller Hinton broth (MHB; BD, 211443) for the bacterial adhesion tests. The suspension was diluted to the target inoculum concentration using optical density (OD) measurements at 600 nm. The target inoculum was serially diluted, and 100 µL aliquots were plated in duplicate on blood agar plates to quantify the exact colony-forming units (CFU)/mL.

#### Antibacterial activity test.

The antibacterial activity of the coating was tested using ISO 22196, ASTM E2180-18, and JIS Z 2801 standards [27–29], as described by van Hoogstraten et al. [31]. The experiment involved three coated and six uncoated samples of both Ti and UHMWPE for each bacterial strain. Samples were prewetted with 0.85% saline using a cotton bud, and a 3 mg/mL agar slurry (pH 6.8-7.2) (Sigma-Aldrich, 05040) was prepared. *S. aureus* ATCC6538 and *E. coli* ATCC8739 overnight cultures were diluted to 1.0 × 10^5^ CFU/mL with the agar slurry at 45°C. For Ti samples, 100 µL of the slurry was applied. For the more hydrophobic UHMWPE samples, the slurry was first diluted 1:2, and 200 µL was used to ensure complete surface coverage. The slurry was gelated on the samples. Three uncoated Ti and UHMWPE samples were processed immediately to determine the starting inoculum. The remaining samples were incubated for 24 h at 37°C. For processing, samples and agar slurry were placed in 5 mL neutralizing broth (casein peptone lecithin polysorbate broth, Sigma-Aldrich, 22089; Tween-20, Sigma-Aldrich, P7949), sonicated in a water bath (Branson 2210 sonicator, Branson Ultrasonics Co., USA) for 10 minutes, and vortexed for 5 s. The sonicate was serially diluted, and 100 µL aliquots were plated on blood agar plates in duplicate to quantify viable bacteria. CFU were counted after 24 h incubation at 37 °C. Antibacterial activity (*R*) is calculated as the difference in the logarithm of viable cell counts between treated (*A*_*t*_) and untreated (*U*_*t*_) samples [27].

Resulting in the following formula:


$$R=U_{t}-A_{t}$$ (1)

Furthermore, the percentage of CFU reduction between the PEL-10/HA144-1 coated samples and the uncoated samples was calculated.

#### Bacterial adhesion test.

A bacterial suspension test was conducted to evaluate the prevention of bacterial adhesion to the samples by the PEL-10/HA144-1 coating. *S. aureus* ATCC25923 and *E. coli* ATCC25922 overnight cultures were diluted to ~1 × 10^7^ CFU/mL in MHB. Four samples per group were placed in a 24-well plate with 1 mL inoculum and incubated for 4 or 24 h at 37°C and 60 rpm, for each bacterial strain and time point. After washing with 2 mL PBS to remove non-adherent bacteria, three samples per group were sonicated in PBS for 5 minutes, serially diluted, and plated on blood agar plates in duplo to count CFU after 24 h incubation at 37 °C. One sample per group was prepared for scanning electron microscopy (SEM) analysis by fixation in 2.5% glutaraldehyde (Sigma-Aldrich, 8.20603.100) in 0.1 M phosphate buffer (PB) (pH 7.4), followed by three 15 min washes in 0.1M PB, fixation in 1% osmium tetroxide, and a final wash with PB. The samples were dehydrated in ethanol (70% for 30 min, 90% for 30 min, 100% for 1 h), and chemically dried with hexamethyldisilazane (Sigma-Aldrich, 8.04324.0250). Samples were mounted on aluminum stubs, sputter-coated with 5 nm carbon (Leica ACE600), and imaged using SEM (Jeol JSM-IT200 InTouchScopeTM, Germany) to visualize biofilm morphology and bacterial adherence. SEM images were also taken of samples unexposed to bacteria for comparison.

### Biocompatibility and osteogenic function assessment

#### Cell lines and preparation.

All cell lines were stored in liquid nitrogen until use and confirmed to be mycoplasma negative. L929 mouse fibroblasts (ATCC cell line CCL 1, NCTC clone 929) were cultured in supplemented Dulbecco’s modified Eagle’s medium (DMEM/F12 low glucose & GlutaMAX, Invitrogen, Carlsbad, CA, USA) supplemented with 10% Fetal Calf Serum (FCS) and 1% antibiotic antimycotic solution (anti-anti; Gibco, USA). Osteoblast-like cells Saos-2 (CLS, CLS 300331, epithelial-like) were cultured in DMEM Glutamax (Gibco, 61965−026) supplemented with 10% Fetal Bovine Serum (FBS, A5256801) and 1% penicillin/streptomycin (p/s; P4333, Sigma-Aldrich). All cells were cultured at 37°C in a humidified atmosphere containing 5% CO_2_.

#### Cell viability and toxicity.

Test on extracts of the PEL-10/HA144-1 coating was performed according to ISO 10993−5 [32] for cell viability and toxicity, with all experiments in triplicate for both cell lines. Three PEL-10/HA144-1-coated and three uncoated samples of Ti and UHMWPE were placed in a 24-well sterile polystyrene tissue culture plate. Supplemented cell line-specific culture medium was added at 3 cm^2^/mL, in total 1.74 mL per sample, as ISO 10993−5 recommended. 1% Triton X-100 served as a positive control, and medium as a blank control. All extracts and controls were incubated for 72 h at 37 °C and 60 rpm.

After 48 h of sample incubation, cells were seeded in 96-well cell culture plates (1 × 10⁵ cells/mL, 100 µL/well) and incubated for 24 hours at 37 °C, 5% CO₂, reaching 80% confluence. A dilution series of sample extracts (100%, 50%, 25%, 12.5%) was prepared. The medium was aspirated from the cells and replaced with 100 µL of extract dilutions or controls, followed by a 24-hour incubation at 37 °C, 5% CO₂. Six technical repeats were conducted for all dilutions and controls. Post-incubation, morphological changes were microscopically evaluated. Water-soluble tetrazolium salt (WST-1; Sigma-Aldrich, 11644807001) and lactate dehydrogenase (LDH; Sigma-Aldrich, 11644793001) assays were used for quantitative evaluation. The medium was aspirated, of which 50 µL was transferred to a new 96-well plate for the LDH assay. Cell line-specific culture medium with 10% WST-1 was prepared; 100 µL of WST-1 supplemented medium was added to each well and incubated for 2−3 hours at 37 °C, depending on the cell line (3h for L929, 2h for Saos-2). Absorbance was measured at 450 nm (*A450*) and 650 nm (*A650*) for test and control samples. The viability was calculated as follows:


$$Viability\;\%=\;\frac{\text{1}\text{0}\text{0}\cdot {(A}_{\text{4}\text{5}\text{0},test}{-A}_{\text{6}\text{5}\text{0},test})}{{(A}_{\text{4}\text{5}\text{0},blank}{-A}_{\text{6}\text{5}\text{0},blank})}$$ (2)

The ISO 10993−5 testing standard defines a reduction of cell viability >30% as a cytotoxic effect [32].

To support the WST-1 assay results, an LDH assay was conducted to evaluate cytotoxicity. The LDH reaction mixture was prepared per the manufacturer’s instructions. A standard curve of nicotinamide adenine dinucleotide (NADH) solutions (0 to 0.75 µmol/mL) was created. 50 µL of the LDH reaction mixture was added to 50 µL of the sample and NADH solutions and incubated at room temperature in the dark. Absorbance was measured at 492 nm after 2–3 minutes and 30 minutes. LDH activity was calculated using the NADH standard curve, following the manufacturer’s instructions. Cytotoxicity is compared to the 1% Triton X-100 as a positive control as follows:


$$Cytotoxicity\;\%=\;\frac{\text{1}\text{0}\text{0}\cdot {LDH}_{t}}{{LDH}_{PC}}$$ (3)

With *LDH*_*t*_ as the LDH activity of the test sample and *LDH*_*PC*_ as the LDH activity from the positive control.

#### Direct contact viability of osteosarcoma Saos-2 cells.

PEL-10/HA144-1 coated and uncoated Ti samples were placed in a non-treated 24-well polystyrene plate and incubated for 24 h with full medium (DMEM + 10% FBS + 1% p/s). Saos-2 cells, grown to passage 10, were seeded on the substrates at 10,000 cells/cm² density. A cell culture-treated polystyrene 24-well plate served as a control. Cells were cultured for up to 21 days, with viability assessed on days 1, 3, and 7 using WST-1, LDH, and bicinchoninic acid (BCA) assays. 100 µL supernatant was collected for LDH analysis, and µg LDH was calculated using a LDH standard curve. 500 µL fresh medium with WST-1 reagent was added for 1-4h incubation, depending on the time point. 100 µL aliquots of WST-1 solution were measured at 440 nm absorbance with 620 nm as a reference. Cells were collected using Accutase, lysed via freeze/thaw cycles in 0.1% Triton X, and analyzed for protein content using the Pierce BCA Protein Assay Kit (Ref 23225), following the manufacturer’s protocol. WST-1 and LDH results were normalized to protein content to correct for attachment differences between conditions. The normalized WST-1 and LDH were used to determine metabolic activity per cell and cytotoxicity, respectively.

#### Attachment of osteosarcoma Saos-2.

Cell morphology on coated and uncoated materials was examined using fluorescent staining. On day 1, samples were fixed with 4% PFA, permeabilized with 0.1% Triton X, and stained with DAPI and Phalloidin 488. CellProfiler 4.2.6 was used to quantify the nucleus and cytoskeleton area in the fluorescent images.

#### Osteogenic markers of osteosarcoma Saos-2.

From day 3 onwards, cells were exposed to osteogenic medium, full cell culture medium supplemented with 10^−8^ M Dexamethasone (D4902, Sigma-Aldrich), 10 mM ß-glycerophosphate (G9422, Sigma-Aldrich), and 0.2 mM L-ascorbic acid (A2174, Sigma-Aldrich), to stimulate osteoblast differentiation and mineralization. To assess Osteoprotegerin (OPG) levels, from day 3 onwards, cells were further stimulated with osteogenic medium supplemented with 10^−7^ M 1α,25-dihydroxyvitamin D3. OPG levels in culture supernatant were evaluated using the Human OPG Instant ELISA Kit (Invitrogen, BMS2021INST), with samples collected on day 6 and analyzed according to the manufacturer’s instructions. Results were normalized to protein content on day 7. On day 21, the mineralization ability of the cells was assessed. Cells were fixed with 4% PFA, washed with DPBS, and stained with 2% Alizarin Red. Quantification was done based on the protocol of Gregory et al. [33]. Samples were carefully washed, followed by adding 10% acetic acid for 30 min with gentle shaking. The solution was then collected and incubated for 10 min at 85°C and 750 rpm. Next, it was centrifuged at 13,000 g, and 500 µL of total volume was extracted and neutralized with 200 µL of 10% ammonium hydroxide. Absorbance was measured at 405 nm. This assay is considered a semi-quantitative assay.

### Statistical analysis

Statistical analysis was performed using GraphPad Prism 10.1.2 (GraphPad Software, Inc.) for Windows. The Shapiro-Wilk test was performed to check if the data was normally distributed. The results from the PEL-10/HA144-1 coated and the uncoated samples were compared per bacterial strain and substrate material with an unpaired t-test for the antibacterial activity test when the data was normally distributed. When data was not normally distributed, a Mann-Whitney U-test was used. To calculate the log difference between the coated and uncoated samples, the logarithm of all values was computed. These logarithmic values were then averaged, generating the geometric mean, and the mean of the coated samples was subtracted from the mean of the uncoated samples. One-way ANOVA with multiple comparisons was used for the biocompatibility assay when the data was normally distributed. When the data was not normally distributed, a non-parametric Kruskal-Wallis test was used. For the WST-1 and LDH assays, the results from the uncoated samples were compared to the PEL-10/HA144-1 coated samples. Furthermore, for the WST-1 assay, the untreated cells were compared to all other results. P-values <0.05 were considered statistically significant. For the osteoblast direct contact tests, a two-way ANOVA was used, and normal distribution was assumed with n = 3.

## Results

### Coating characterization

**Fig 2:**
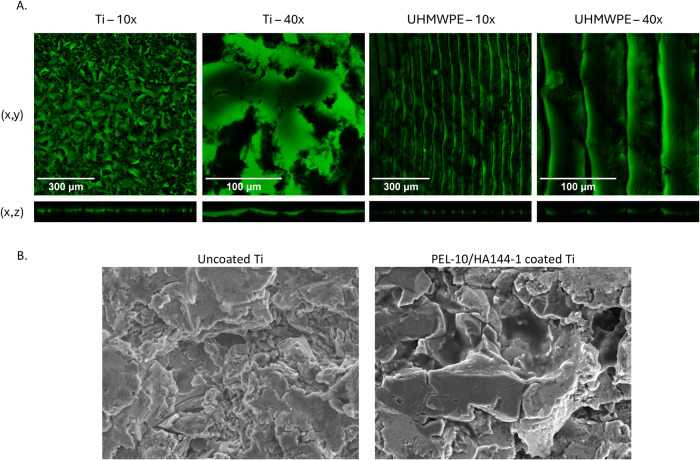
PEL-10/HA144-1 coating visualization. PEL-10/HA144-1 coating labeled with Poly-L-Lysine coupled with fluorescein isothiocyanate (PLL-FITC) (green) was observed with confocal microscopy on both titanium (Ti) and ultra-high-molecular-weight-polyethylene (UHMWPE) samples **(A)**. Scanning Electron Microscopy (SEM) images of Ti samples without (left) and with (right) PEL-10/HA144-1 coating **(B)**. SEM settings: 10.0kV, WD10mm, PC 40.0, 3000x.

**Fig 3:**
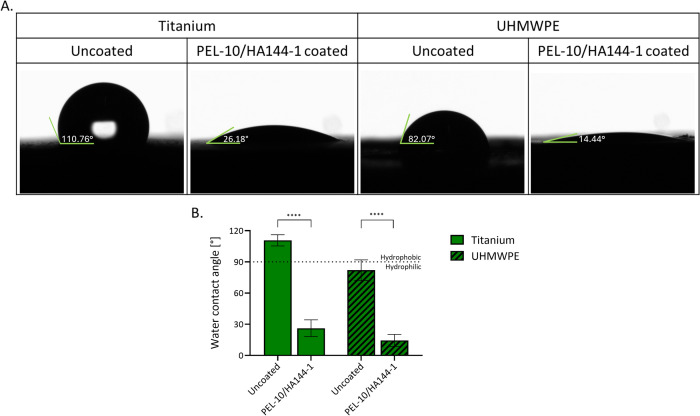
The water contact angle (WCA). Visualization of the WCA of titanium and UHMWPE substrates, either uncoated or coated with PEL-10/HA144-1 **(A)**. The mean of the WCA of three locations on three samples is displayed ±SD **(B)**. Statistical differences found with an unpaired t-test are presented with ****P < 0.0001.

To characterize the basic parameters of the coating, confocal microscopy was used to visualize the coating, the water contact angle was measured, and the roughness and a POD wear test were performed. As visualized with confocal microscopy and PLL-FITC labeling, the PEL-10/HA144-1 coating was deposited onto the Ti and UHMWPE substrates (Fig 2A). Both confocal microscopy and SEM imaging demonstrate that the coating molds to the structure of the material beneath it (Fig 2). Fluorescence is bright, indicating a homogeneous coating. With the SEM images, it is visualized that the sample was coated completely, and that it slightly affects the coating roughness. The uncoated samples demonstrated an irregular and rough surface morphology, while the PEL-10/HA144-1 coated samples were slightly smoother. The WCA can range from 0° to 180°, with >90° being hydrophobic, and <90° being hydrophilic. The WCA for the unmodified Ti was hydrophobic (110.76°) and changed to hydrophilic by coating with PEL-10/HA144-1 (26.18°). Unmodified UHMWPE had a hydrophilic WCA (82.07°), which lowered further after coating with PEL-10/HA144-1 (14.44°) (Fig 3). The mean wear rate of the PEL-10/HA144-1 coating (average of 7.51 ± 1.06 mg at 0.99 million cycles (MC)) samples articulated on CoCr disks was not statistically significantly different from the mean wear rate of the control pins (average of 6.64 ± 0.30 mg at 0.99 MC) tested on CoCr disks (p = 0.472). The roughness of the coated samples (1.454 ± 0.106 µm) compared to the uncoated samples (1.543 ± 0.218 µm) did not demonstrate a significant statistical difference between both groups.

### Antibacterial properties

#### Antibacterial activity test.

**Fig 4:**
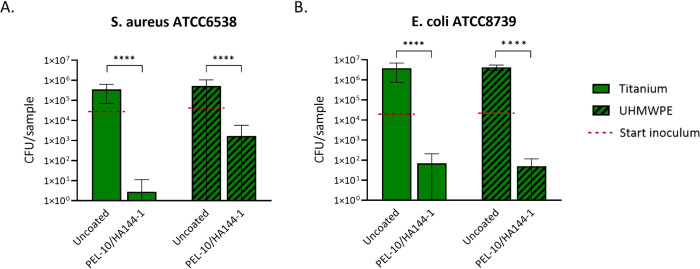
Results from the antibacterial activity test. After 24h of incubation with S. aureus (A) and E. coli (B), viable bacteria were collected from the samples. The starting inoculum is indicated by a red dotted bar. Green bars represent Titanium samples, and green/black striped bars represent UHMWPE samples. Each bar represents the average ± SD of three experiments, each including three samples. Statistical differences found with an unpaired t-test are presented with ** p < 0.01, and ****P < 0.0001. Each bar represents the average ± SD of three experiments, each including three samples. Statistical differences found with an unpaired Mann-Whitney U-test are presented with ****P < 0.0001.

The antibacterial activity of the coating was tested using ISO 22196, ASTM E2180-18, and JIS Z 2801 for regulatory compliance. The average *S. aureus* ATCC6538 starting inoculum was 2.84 × 10^4^ CFU/sample for Ti and 4.37 × 10^4^ CFU/sample for UHMWPE. The average *E. coli* ATCC8739 starting inoculum was 2.49 × 10^4^ CFU/sample for Ti and 2.20 × 10^4^ CFU/sample for UHMWPE. To determine the bactericidal effect after 24 h of incubation, viable bacteria in the agar slurry on coated and uncoated Ti and UHMWPE substrates were counted (Fig 4). Uncoated samples showed a higher CFU/sample compared to the starting inoculum, and the coated samples showed a decrease in CFU/sample compared to the starting inoculum, demonstrating a bactericidal effect of the coating. For *S. aureus* ATCC6538, the PEL-10/HA144-1 coating resulted in a significant reduction of 5.3 and 3.6 log compared to uncoated Ti and UHMWPE, respectively (Table 1), which is a > 99% reduction in CFU. For *E. coli* ATCC8739, the PEL-10/HA144-1 coating resulted in a significant decrease of 5.5 and 5.7log compared to uncoated Ti and UHMWPE, respectively (Table 1), which is a > 99% reduction in CFU.

**Table 1:** Antibacterial activity R and reduction in CFU (%). Induced by the PEL-10/HA144-1 coating in the antibacterial activity test.

Bacterial strain	Substrate	Antibacterial activity R	Reduction in CFU (%)	P value
*S. aureus* ATCC6538	Titanium	5.259	99.999	0.0018
	UHMWPE	3.597	99.683	0.0090
*E. coli* ATCC8739	Titanium	5.453	99.998	0.0018
	UHMWPE	5.708	99.999	<0.0001

#### Bacterial adhesion test.

**Fig 5:**
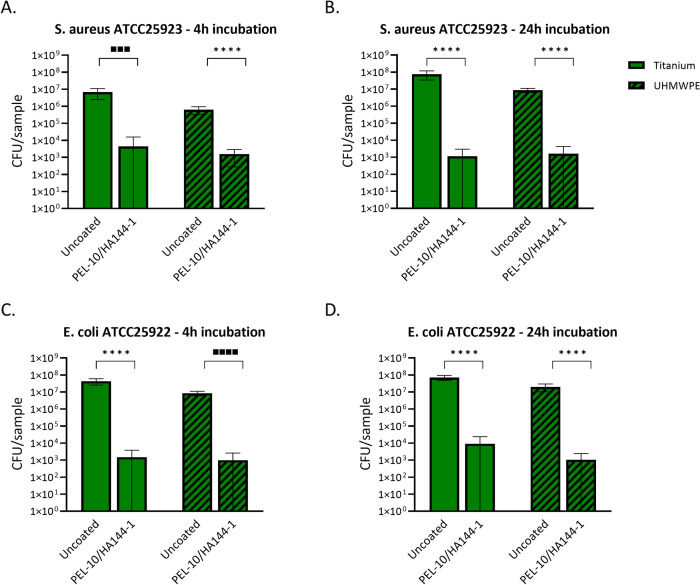
Results from the bacterial adhesion test. Viable bacteria, S. aureus (A and B) or E. coli **(C and D)**, that adhered to the samples after 4h (A and C) or 24h (B and D) of incubation, were collected from the samples. The starting inoculum was 2.23 × 10^7^ CFU/mL and 3.28 × 10^7^ CFU/mL for S. aureus ATCC25923 and E. coli ATCC25922, respectively. Green bars represent titanium samples, and green/black striped bars represent UHMWPE samples. Each bar represents the average ± SD of three experiments, each including three samples. Statistical differences found with an unpaired t-test are presented with ****P < 0.0001. Statistical differences found with an unpaired t-test are presented with ■■■ = 0.0002 and ■■■■ < 0.0001. Statistical differences found with a Mann-Whitney U-test are presented with ****P < 0.0001.

**Fig 6:**
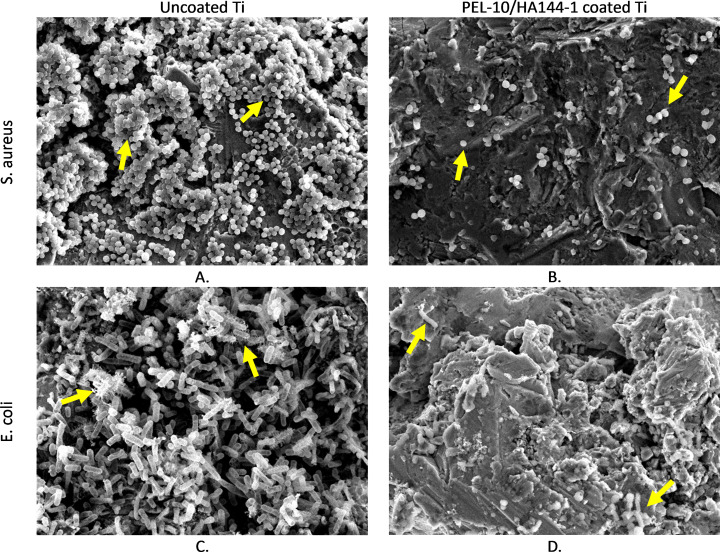
Scanning Electron Microscopy images. Titanium (Ti) samples after 24h incubation in a bacterial adhesion test with S. aureus **(A and B)**, and E. coli **(C and D)**, without (A and C) and with **(B and D)** PEL-10/HA144-1 coating. Settings: 10.0 kV, WD 10 mm, PC 40.0, 3000x zoom. C and E) arrows indicate examples of accumulation of bacteria and biofilm structures; D and F) arrows indicate examples of single S. aureus and E. coli bacteria that are left.

To further investigate the effect of the PEL-10/HA144-1 coating, bacterial adhesion after 4 h and 24 h of incubation was investigated. The starting inoculum was 2.23 × 10^7^ CFU/mL and 3.28 × 10^7^ CFU/mL for *S. aureus* ATCC 25923 and *E. coli* ATCC 25922, respectively. Viable bacteria were retrieved from the samples after 4 h and 24 h incubation with *S. aureus* ATCC25923 and *E. coli* ATCC25922 suspension (Fig 5). The CFU rose from 4 h to 24 h for both bacterial strains on the Ti and UHMWPE uncoated samples. Whereas for the PEL-10/HA144-1 coated samples, the number of viable bacteria after 4 h and 24 h of incubation remained similar, resulting in a higher log10 reduction for 24 h compared to 4 h, except for PEL-10/HA144-1 coated Ti in the *E. coli* ATCC25922 suspension. When looking at the log difference when calculated with the geometric mean, which is skewed less, all coated samples had a > 3 log reduction compared to the uncoated samples (Table 2). SEM images showed clear clusters of bacterial cell populations on Ti samples after 24 hours of incubation on the uncoated samples (Fig 6), whereas the coated samples only showed single bacteria.

**Table 2:** Log10 and percentage reductions between the PEL-10/HA144-1 coated samples and the uncoated samples in the bacterial adhesion test.

Bacterial strain	Incubation time	Titanium			UHMWPE		
		Log10 (±SD) reduction	Reduction in CFU (%)	P value	Log10 (±SD) reduction	Reduction in CFU (%)	P value
*S. aureus* ATCC25923	4h	4.57	99.933	0.0002	3.12	99.751	< 0.0001
	24h	5.34	99.998	< 0.0001	4.13	99.981	< 0.0001
*E. Coli* ATCC25922	4h	5.31	99.997	< 0.0001	4.83	99.988	< 0.0001
	24h	4.37	99.987	< 0.0001	4.58	99.995	< 0.0001

### Biocompatibility and osteogenic function assessment

#### Cell viability and toxicity with L929 and Saos-2 cells.

**Fig 7:**
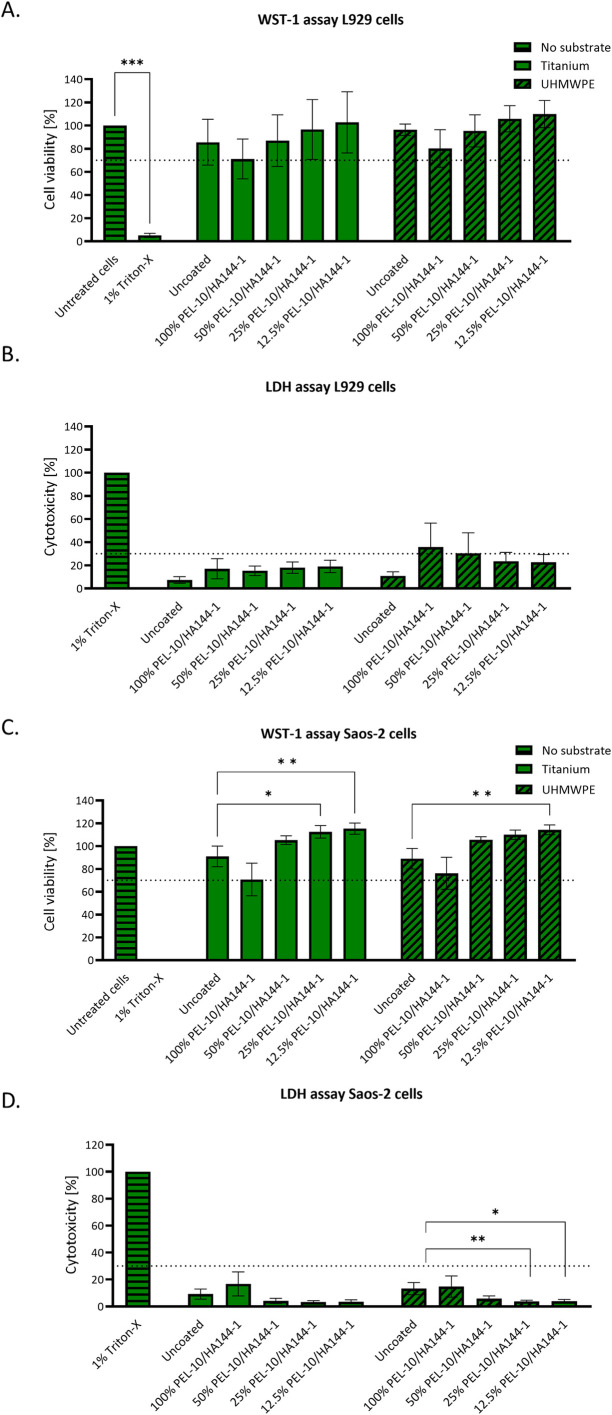
Cell viability [%]. As found by the WST-1 assay for the L929 cells (A) and Saos-2 cells **(C)**, and corresponding cytotoxicity as found by the LDH assay (B for L929 and D for Saos-2 cells). Green bars represent Titanium samples, and green/black striped bars represent UHMWPE samples. Each bar represents the average ± SD of three experiments, each including three samples. Significant differences as found by the Kruskal-Wallis multiple comparison test are displayed in (A) and (C) for untreated cells versus all other samples, and in all graphs for the uncoated sample extract versus all percentages of the coated samples. *p < 0.05, **p < 0.01, ***p < 0.001.

As the safety and antimicrobial function of a coating are often intertwined, the next aim was to gain more insight into the effect of the coating on the *in vitro* host tissue response. For this, first, the ISO 10993−5 standard was applied, describing the *in vitro* cytotoxicity assessment for medical devices, serving as an early screening for leaching of toxic components. Relative cell viability of ≥70% of the untreated cells for the highest concentration of extract is considered non-cytotoxic [32]. Cell viability of ≥70% for all concentrations of the PEL-10/HA144-1 coated sample extracts was observed using the WST-1 assay for both L929 and Saos-2 cells (Fig 7A and C). Furthermore, no significant statistical difference was observed between the cell viability of Ti and UHMWPE uncoated samples compared to the PEL/HA-coated samples. For the Saos-2 cells, the cell viability of the most diluted coating extracts was even significantly higher than that of the extracts from the uncoated samples. No statistical differences were found between the untreated cells, uncoated sample extracts, and coated sample extracts. Cytotoxicity measured with the LDH assay remained <30% for all extracts, except the 100% extract of PEL-10/HA144-1 coated UHMWPE (Fig 7B and D). For the L929 cells, the LDH assay demonstrated no statistically significant differences between the extracts of the uncoated and coated samples. For the Saos-2 cells, the cytotoxicity of the 25% and 12.5% extracts of the coating on UHMWPE was significantly lower than that of the uncoated UHMWPE extract. Based on ISO 10993−5, the PEL-10/HA144-1 coating does not exert an *in vitro* cytotoxic effect on L929 and Saos-2 cells.

#### Attachment of osteosarcoma Saos-2.

While the in vitro cytotoxicity assays were based on material eluates, initial direct cell-material interactions were evaluated by seeding osteoblast-like Saos-2 cells onto uncoated Ti and PEL-10/HA144-1 coated Ti surfaces.

**Fig 8:**
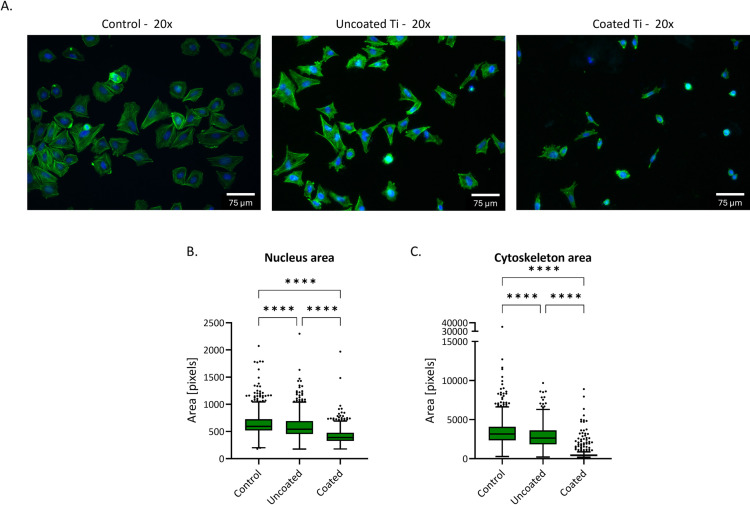
Fluorescent staining of nucleus (DAPI) and actin (Phalloidin) in Saos-2 osteoblast-like cells (A). Cells have been cultured for 24 h on polystyrene control, uncoated Ti, and coated Ti. Standard morphology is observed in the control group, where the morphology of the cells on the uncoated Ti is slightly different. The morphology of the cells on the coated Ti is distorted compared to the control. Fluorescent images (10x magnification) were analysed in CellProfiler to quantify the nucleus **(B)** and cytoskeleton **(C)** differences between conditions. The area was calculated by identifying objects in the pictures, their size, and the number of pixels. The spread of data points is large due to the difference in the size of the cells. Significant differences can be observed between all conditions for the nucleus and cytoskeleton area, as can be observed in Fig 8A. Data represents one experiment with three technical replicates. ****p < 0.0001. Whiskers and outliers are plotted with the Tukey method.

Early attachment was assessed through fluorescent imaging 24 h post-seeding, allowing visualization of cell morphology and adherence (Fig 8A). The uncoated Ti facilitated cell attachment, displaying a morphology comparable to the plastic control, which indicated favorable initial adhesion. In contrast, cells on the coated Ti exhibited a distorted cytoskeletal structure, suggesting that the surface properties of the coating influenced cellular morphology and spreading. Quantification of the fluorescent images with CellProfiler (Fig 8B and C) confirmed this difference by presenting a significantly smaller nucleus and cytoskeleton area in cells attached to the coated Ti compared to uncoated Ti.

**Fig 9:**
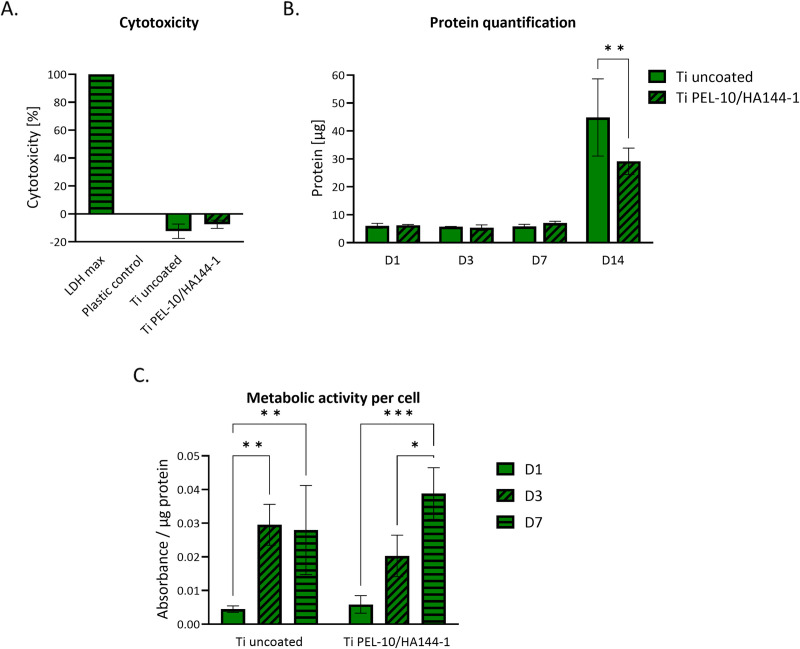
Cell viability of Saos-2 osteoblast-like cells assessed by direct contact. With coated and uncoated Ti discs, the control is a polystyrene well plate. **A)** LDH measurements at day 1, where LDH max was treated with 1% Triton X. **B)** Protein quantification over 14 days. The first week shows no statistical difference between Ti and Ti coating. Additionally, the µg proteins only increased after day 7. At day 14, a statistical difference between Ti and Ti coating can be observed. **C)** Metabolic activity normalized to protein content on day 1, 3, and 7. *p < 0.05, **p < 0.01, ***p < 0.001.

To further assess the early cellular response, a combination of assays was performed over the first week, including LDH assay for cytotoxicity, BCA assay for total protein quantification, and WST-1 assay for metabolic activity per cell. Cell death on day 1, measured by the LDH assay, indicated no immediate cytotoxicity, as levels in the coated and uncoated samples were comparable to the plastic control (Fig 9A). Based on protein quantification, during the first 7 days, cell proliferation appeared to be minimal for coated and uncoated Ti (Fig 9B). However, by day 14, an increase in protein content was observed, suggesting continued cellular activity on coated and uncoated Ti over time. Over 7 days, metabolic activity per cell increased on the coated Ti, suggesting acclimatization to the coated surface (Fig 9C). In contrast, cells on uncoated Ti exhibited a metabolic plateau after day 3. While both coated and uncoated Ti supported initial adhesion without significant cytotoxicity, the differences in cytoskeletal organization, metabolic trends, and proliferation dynamics suggest that the coating alters the early cellular response.

#### Osteogenic markers of osteosarcoma Saos-2.

**Fig 10:**
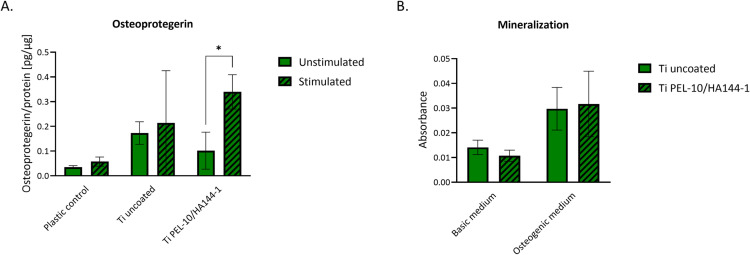
The osteoblast phenotype of Saos-2 cells, assessed using mineralization and secretion of Osteoprotegerin. **A)** A significant difference is observed in the secreted Osteoprotegerin per cell between control and stimulated conditions for the coated Ti. **B)** The mineralization assay shows that cells can mineralize in the presence of the coating while stimulated with osteogenic medium. The stimulated conditions are osteogenic medium supplemented with 1α,25-dihydroxyvitamin D3. *p < 0.05.

The osteoblast-like Saos-2 cells were also evaluated for their osteogenic characteristics, which are essential for determining their functional behavior on the material surfaces. The cells were stimulated with an osteogenic medium to promote an osteocyte-like phenotype, inducing matrix mineralization, and osteogenic medium supplemented with 1α,25-dihydroxyvitamin D3. On day 6, no significant differences in OPG secretion, a protein involved in bone remodeling, were detected for the polystyrene wells and uncoated Ti between the samples stimulated with osteogenic medium supplemented with 1α,25-dihydroxyvitamin D3 and the unstimulated baseline condition (Fig 10A). The coated Ti shows a significant elevation of OPG for the stimulated compared to the unstimulated condition, indicating the ability of the cells to support bone formation in the presence of the coating. Mineralization was assessed after 21 days of culture on uncoated and coated Ti surfaces (Fig 10B). Calcium deposition was observed on coated and uncoated Ti, demonstrating that both materials support the osteogenic potential of Saos-2 cells.

## Discussion

PJI remains a major clinical problem following THA and TKA, resulting in high treatment costs and a diminished patient’s quality of life [16]. Due to the protective barrier formed by biofilms and rising antimicrobial resistance against antibiotics, therapeutic insufficiency is an increasing problem for patients with a PJI [8,12,13]. This study investigated the antibacterial activity of a novel polyelectrolyte contact-killing spray coating, based on the polycation Poly-ε-Lysine (PEL-10) and the polyanion hyaluronic acid (HA144−1). A broader biological evaluation was conducted by assessing biocompatibility and relevant biological processes, integrating a development perspective through a Safe-by-Design approach with *in vitro* tests for implant-host interactions.

The mode of action of the PEL-10/HA144-1 coating, combined with the material surface characteristics, plays an important role in preventing bacteria’s initial adhesion [34]. HA creates a hydrophilic hydration layer on the substrate, inhibiting protein absorption and resisting bacterial adhesion due to its antifouling properties [14,24]. Furthermore, the coating significantly reduced the WCA. A low WCA is advantageous for the coating’s contact-killing mechanism as it enhances the electrostatic interaction between the PEL-10 in the coating and the bacteria [35]. As observed by both antibacterial activity tests, the PEL-10/HA144-1 coating significantly reduced bacterial adhesion on both Ti and UHMWPE surfaces.

Two tests were utilized to assess the coating’s antimicrobial efficacy in different conditions. Antibacterial efficacy against the gram-positive *S. aureus* and gram-negative *E. coli* was first tested using an antibacterial activity test [27–29]. PEL-10/HA144-1 coated samples showed significant log reductions in CFU compared to the initial inoculum and uncoated samples, demonstrating a potent bactericidal effect. Compared to another study, combining several studies from different laboratories, the R-values acquired in the current work demonstrate a significant antibacterial effect of the PEL-10/HA144-1 coating for *S. aureus* on UHMWPE and a strong antimicrobial effect for *S. aureus* on Ti, as well as *E. coli* on both Ti and UHMWPE [36]. Typically, a 2−3 log, or 99-99.99%, reduction in surface-adherent CFU is considered a clinically relevant effect for antimicrobial compounds [29,37–39]. However, this threshold may not account for the bacteria’s rapid regrowth and adaptive capabilities *in vivo* [29,37–39]. The PEL-10/HA144-1 coating demonstrated a promising antibacterial effect *in vitro*, which is likely clinically relevant. Based on these standard test results, the step to *in vivo* testing can be made.

The standard tests were corroborated with an adhesion test in a more challenging environment. Moreover, each bacterial strain can differ in, e.g., its virulence or biofilm formation [40]. Therefore, other *S. aureus* and *E. coli* strains were used than those specified in the standard test methods, typically utilized for testing antimicrobial compounds *in vitro*. Despite these challenging conditions, a significant and clinically relevant reduction of adherent bacteria was observed for the PEL-10/HA144-1 coated samples compared to the uncoated samples. While the CFU increased over time for the uncoated samples, the CFU remained relatively stable for the coated samples, demonstrating a sustained bacteriostatic effect for both *S. aureus* and *E. coli*. The SEM images confirmed reduced bacterial presence and biofilm formation for the coated compared to the uncoated samples. The combination of the ISO standard and adhesion tests allowed for an elaborate assessment of the antimicrobial properties of the coating, demonstrating its bacteriostatic and bactericidal effect.

The clinical relevance of these *in vitro* results remains to be demonstrated for both antibacterial tests, as not all bacteria were eradicated, which might proliferate or disperse. Multiple studies have shown significant and clinically relevant reductions in CFU *in vitro*; however, this did not necessarily translate to *in vivo* studies due to the complex host environment, and possibly because the contact-killing coating did not account for dispersed bacteria [31,41–43]. As the intended use is preventing PJI, and not treating it, the inoculum levels tested in this experiment were substantially greater than those typically encountered in the clinical setting [38]. As the coating demonstrated both bacteriostatic and bactericidal effects, future research is essential to elucidate the mechanism of action. Test conditions and coating concentration are known to alter these effects [39].

With cell viability above 70%, the PEL-10/HA144-1 coated sample extracts exhibited limited *in vitro* cytotoxicity against L929 fibroblast and Saos-2 osteoblast-like cells, according to ISO 10993−5 [32]. As the cell type has been shown to affect the results, both L929 and Saos-2 cells were utilized in the cytotoxicity assessment [44]. L929 cells to adhere to ISO standards, and Saos-2 cells to more accurately reflect the cells surrounding an orthopedic implant. For both cell lines, viability increased with lower extract concentrations, indicating a cellular response to the PEL-10/HA144-1 coating. The 72 h incubation for extract preparation, as specified by ISO 10993−12 [45], does not accurately reflect *in vivo* conditions, where fluid circulation transports substances from the implantation site [44]. However, it is an essential first chemical screening for biological evaluation. Although a trade-off between antibacterial activity and biocompatibility is common, the PEL-10/HA144-1 coating maintains cell viability and toxicity within acceptable limits [14,46]. HA and PEL have already demonstrated good biocompatibility according to ISO 10993−5. These polymers are naturally occurring, biodegradable, and are already used in clinical and food applications [20,24,47,48]. Therefore, high cell viability of the PEL-10/HA144-1 coating was expected.

Evaluation of the impact of the coating on the osteoblast response is essential in a safety assessment. Therefore, Saos-2 adhesion, proliferation, and osteogenic markers were assessed over a three-week culture period. The electrostatic interactions from the PEL-10/HA144-1 coating that make the coating contact-killing might affect the osteoblast microenvironment. Chemical and physical stimuli from the osteoblast microenvironment dictate osteoblast behavior, and possibly affect osseointegration [49]. Fluorescent imaging demonstrated a distorted cytoskeleton, suggesting a challenging initial attachment. Which could be explained by the lack of attachment sites of the polymer HA [50]. However, a positively charged surface, due to the excess of PEL-10 in the coating, is favored by cells for adherence [51]. During the first week, whereas metabolic activity remains stable after day 3 for the uncoated Ti, the cells gradually increase their metabolic activity on the coated Ti, possibly due to degradation of the coating. Notably, the coating does not inhibit osteoblast proliferation and mineralization. Further research is needed to assess the mechanism behind the decreased initial osteoblast attachment and metabolic activity due to the coating.

For optimal implant performance, the implant and coating design must account for key interacting biological processes, in alignment with the SbD principles [18]. SbD involves considering risks and uncertainties related to human safety, early in the innovation and development stages [52,53]. For orthopedic implant technologies, the prevention of bacterial adhesion, enabling osseointegration, and immune acceptance are crucial for successful implementation [18]. Assessing all three biological processes will allow for a balanced SbD evaluation on safety and functionality, guiding future *in vivo* research [18]. While ISO standards offer a standardized framework for assessing implant safety and efficacy, these *in vitro* methods often lack complexity, restricting their ability to predict *in vivo* translation. Therefore, this study implemented more advanced *in vitro* methods, which can serve as an insightful pre-screening tool for biological processes before *in vivo* testing, increasing translational success and patient safety by offering more robust data on safety and functionality.

This research expanded the standard *in vitro* assessment by adding *in vitro* tests for bacterial adhesion, cell viability, and osteoblast phenotype in a direct contact setup. The results indicate that the coating can kill bacteria but also highlight the need for increased investigation of initial bone integration. Moreover, defining a threshold for antimicrobial coating efficacy is challenging as (standard) testing methods vary, and the *in vitro* log reduction may not translate to *in vivo* [54]. Variables such as incubation time, medium dilution, inoculum size, and bacterial growth phase all affect the test [36]. Moreover, these tests lack the complexity of *in vivo* systems [37]. Similarly, for ISO 10993−5 standard-based biocompatibility testing, the researcher can choose the medium type, percentage of serum, test setup (extract, direct, or indirect contact), and cell type [44]. All these factors influence the test results, complicating comparative analysis or setting a standard baseline. Moreover, the immune response remains a crucial factor in implant success and should be studied *in vitro*, e.g., by testing macrophage polarization over time towards the pro- or anti-inflammatory phenotype.

The multifaceted *in vitro* assessment of relevant biological processes provided a comprehensive and realistic basis for advancing to a preclinical *in vivo* animal study for the evaluation of the PEL-10/HA144-1 coating.

This study is crucial for examining host response, implant integration, and pathogen interaction within a closed system, potentially helping to bridge the translational gap to clinical application [55]. While initial *in vitro* testing is essential, site-specific *in vivo* models are crucial because they evaluate the performance of antimicrobial coatings in the complex, realistic conditions of the actual implantation site [43].

## Conclusion

This comprehensive evaluation explored the potential of the PEL-10/HA144-1 coating technology for the prevention of bacterial adherence following TKA and THA, with no direct concerns for biocompatibility and functional implant-tissue interaction based on *in vitro* data. The coating was successfully deposited on Ti and UHMWPE substrates. The coating demonstrated effective bacteriostatic and bactericidal effects, with clinically significant CFU reductions when compared to uncoated substrates. Moreover, cell viability for both L929 and Saos-2 cells remained acceptable. Despite the lower initial attachment of Saos-2 cells, the coating supported osteoblast function and maintained osteogenic marker expression over more extended periods. These results provide essential information for SbD application in early innovation steps of the PEL-10/HA-144-1 coating, helping to balance safety considerations to prevent bacterial adhesion while maintaining osteoblast response. These *in vitro* findings support the PEL-10/HA144-1 coating’s advancement to pre-clinical *in vivo* evaluation.

## Supporting information

S1 File Data sets for all samples are available in DataverseNL under https://doi.org/10.34894/IDSQ84. (XLSX)

S2 File Graphical abstract. (TIF)
