# Supplementary figures and images for: In vitro assessment of antibacterial and biocompatibility properties of a poly-ε-lysine and hyaluronic acid contact-killing coating to prevent prosthetic joint infection
Source: PLoS One. 2026 Jan 30;21(1):e0340632. doi: 10.1371/journal.pone.0340632 (PMC12857985; doi:10.1371/journal.pone.0340632)

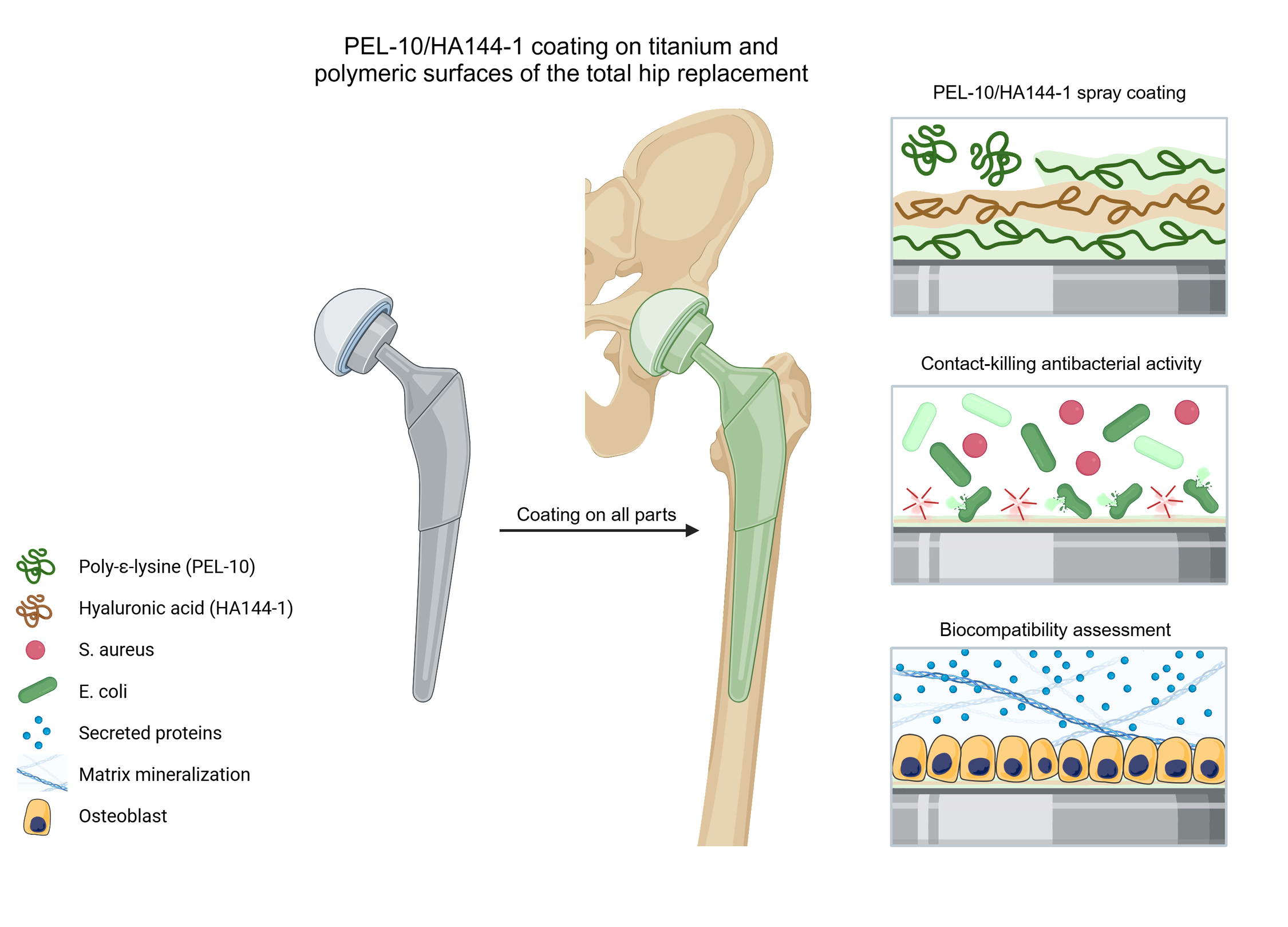

Supplement: S2 File — (TIF) [file pone.0340632.s002.tif]
